# The Spanish HIV BioBank: a model of cooperative HIV research

**DOI:** 10.1186/1742-4690-6-27

**Published:** 2009-03-09

**Authors:** Isabel García-Merino, Natividad de las Cuevas, José Luis Jiménez, Jorge Gallego, Coral Gómez, Cristina Prieto, Ma Jesús Serramía, Raquel Lorente, Ma Ángeles Muñoz-Fernández, Spanish HIV BioBank

**Affiliations:** 1 Laboratorio de Inmunobiología Molecular, Plataforma de Laboratorio, Hospital General Universitario Gregorio Marañón, Madrid, Spain; 2 Unidad Asociada de Retrovirologia Humana, HGUG-CSIC, Madrid, Spain

## Abstract

**Background:**

The collection of samples from HIV-infected patients is the beginning of the chain of translational research. To carry out quality research that could eventually end in a personalized treatment for HIV, it is essential to guarantee the availability, quality and traceability of samples, under a strict system of quality management.

**Methods:**

The Spanish HIV BioBank was created with the objectives of processing, storing and providing distinct samples from HIV/AIDS patients, categorized according to strictly defined characteristics, free of charge to research projects. Strict compliance to ethical norms is always guaranteed.

**Results:**

At the moment, the HIV BioBank possesses nearly 50,000 vials containing different prospective longitudinal study sample types. More than 1,700 of these samples are now used in 19 national and international research projects.

**Conclusion:**

The HIV BioBank represents a novel approach to HIV research that might be of general interest not only for basic and clinical research teams working on HIV, but also for those groups trying to establish large networks focused on research on specific clinical problems. It also represents a model to stimulate cooperative research among large numbers of research groups working as a network on specific clinical problems. The main objective of this article is to show the structure and function of the HIV BioBank that allow it to very efficiently release samples to different research project not only in Spain but also in other countries.

## Correspondence

Advances in research and technology allow for the design of new experimental approaches to use the same biological specimens that have previously given positive results. It is important not to undervalue biological material that has already undergone studies in the development of new scientific approaches. In Europe and North America, laboratories that store distinct sample types and in some way "function as tissue and biological fluid banks" have been essential parts of AIDS research.

Biobanks are identified as a biomedical scientific/infrastructural development that represents a political/legal/ethical reaction with the goal to integrate Biobanks into the pre-existing form of regulation, medicine law and society[1]. Some basically consider any inventory or file of biological material as a Biobank, whereas in other countries Biobanks are seen as the major research infrastructure. However, each research project using established Biobanks should be preceded by the careful assessment by both the researchers themselves and the Ethical Committee to find out predictable risks and burdens in relation to foreseeable benefits to the subject and others. This assessment must include a consideration of good practice in storing, coding and using samples as well as appropriate procedures for obtaining consent and counseling[1].

The HIV BioBank belongs to the Laboratory Platform, a part of the RED RIS[2], organised by the Laboratorio de Inmuno-Biología Molecular of the Hospital General Universitario Gregorio Marañón. Therefore, a single hospital is responsible for the preservation and storage of these HIV samples.

The primary objective of the Spanish HIV Research BioBank is to contribute to furthering scientific knowledge about HIV infection by providing biological samples from HIV-infected patients that are included in cohorts for the objective of carrying out research. The HIV BioBank receives samples from 28 hospitals, spread across Spain, which are grouped into 6 cohorts of HIV-patients with defined characteristics: Cohort of Adults (CoRIS), Cohort of Long Term Non-Progresors (LTNP), Cohort of Rapid Progressors, Cohort of Acute or Recent Infection, Cohort of Lymphocyte Non-regenerators, and Cohort of HIV-infected patients with liver organ transplant (OLT-HIV).

The HIV BioBank is managed by a scientific director and data manager who are assisted by a Scientific and Ethics Committee. The Scientific Committee, represented by clinical researchers and participating institutions, have created the basic regulations for the Internal Organization of the HIV BioBank and participate in the scientific review of the procedures. An independent Ethics Committee reviews the agreements made with the different cohorts with respect to the patients' informed consent.

To receive samples by the HIV BioBank, the BioBank director and the coordinator of the participating Cohort must sign a "Deposit Agreement" which lays out how the hospital must send the samples and how they are to be processed and stored. A list of the participating hospitals and the personnel involved is included.

The hospital personnel must coordinate with the BioBank to set the date for the sample shipment to the BioBank. The BioBank is responsible for sending the courier service to the hospital to collect the samples and deliver them to the BioBank.

To donate samples, the HIV-individual must sign an informed consent form in which the risks and discomforts associated with obtaining the samples and the research objective for obtaining the samples are clearly explained. This informed consent form must go to the Ethics and Clinical Research Committee and is stored in the Clinical History of the HIV-individual. Throughout this process the official law on protection of personal data is in effect[3,4]. As a general rule, the consent can always be cancelled. If the HIV-individual decides that he does not want his sample to be used for research, his sample will be destroyed. In all cases, the sample bears a numeric code that can be used to remove the associated patient's data. Therefore, data are blinded because the link between the code and the patient identity can be recovered [5].

Once the samples have been deposited, they are processed to obtain the distinct components (blood, serum, plasma, solid or liquid tissue, DNA, RNA, pellet cells, PBMCs for physiological studies) and cryopreserved in the BioBank. The processing is done through the regulated establishment of a specimen storage facility in order to maintain the sample viability for present and future research projects designed to further the understanding of HIV pathology and knowledge of current biotechnology. The HIV BioBank has been set up according to a system of quality management based on the rules written in UNE-EN-ISO 9001:2000.

To assure that the information kept by the BioBank on the stored samples is correct, periodic checks and comparisons of data between the BioBank, the cohorts and the hospitals are carried out.

BioBank samples can be applied for by any researcher who is a member of the AIDS Network, or anyone in collaboration with a member as long as the project is scientifically, technically and ethically viable. To receive samples, a researcher must complete a sample release application. This application must be submitted evaluation by the members of the Scientific Committee. If the project is approved, the researcher signs a Release Agreement with the director of the BioBank and with the coordinator of the Cohort. The BioBank and the Cohort are responsible for locating the type and number of samples needed to carry out the project. Once the samples have been released, the principal researcher sends a scientific report containing their results once a year so that the BioBank can keep up-to-date records on all the projects.

The HIV BioBank does not charge any fee for providing samples to other research groups apart from the cost of transporting the samples under optimal conditions to assure their arrival in the best condition at the laboratory where they are to be used.

At present, the HIV BioBank has more than 50,000 vials containing different sample types from more than 1500 HIV infected patients. Table 1 shows the number of samples deposited in the BioBank obtained from Adult and LTNP cohorts initiated in 2004 and the number of vials of each sample type. All the BioBank samples belong to prospective longitudinal studies.

**Table 1:** Participating patients in sample donation to the HIV Research BioBank and the date of creation and the sample types donated and for each cohort.

**Cohort**		**Creation's date**	**Sample Type**	**# of patients with samples in the BioBank**
Adults^a)^		3rd June 2004	Blood	2,865

LTNPs^b)^		3rd June 2004	Blood	181

Rapid progressors^c)^		7th February 2008	Blood	19

Non-regenerators^d)^		5th February 2008	Blood	12

Recent infections^e)^	90 days of HIV+	7th February 2008	Blood	13
				
	180 days of HIV+			15

OLT-HIV^f)^		24th January 2008	Blood, liver and spleen biopsies	21

a) Cohort of Adults (CoRIS): includes patients older than 13 with a confirmed diagnosis of HIV-infection that have not received prior antiretroviral treatment and are being seen for the first time in the participating center; b) Cohort of Long Term Non-Progresors (LTNP): includes patients who have T-lymphocyte counts > 500/μL and undetectable viremia for more than 10 years; c) Cohort of Rapid Progressors: includes patients whose CD4 levels have fallen below 300 in less than 3 years after seroconversion and with a window of less than 3 years between the last negative and first positive HIV test; d) Cohort of Lymphocyte Non-Regenerators: includes patients who began HAART with T-lymphocyte < 200/μL that remained under observation with high activity antiretroviral therapy (HAART) treatment during a period of at least 2 years during which the viremia was consistently undetectable. At the conclusion of the observation period, their CD4 values must not have reached the critical level of 250 cells/μl and e) Cohort of Recent Infection: patients with a documented date of seroconversion less than 6 months prior; f) Cohort of HIV-infected patients with organ liver transplant (OLT-HIV): includes HIV-infected patients that have received a liver transplant primarily as a consequence of hepatitis C virus (VHC) induced cirrhosis.

At the moment, the samples deposited in the HIV BioBank have been used in 19 national and international research projects. In general, the projects fall into several categories: a) study and characterization of polymorphisms in the genes of HLA, chemokines, cytokines, immune recognition, and antigen processing; b) comparative studies with diverse qualitative parameters on the anti-HIV immune response measured by CD8+ cells; c) identification of cellular cofactors which could represent antiviral targets; d) studies on viral tropism, envelope cytopathicity, capacity to replicate, and detection of restriction factors such as virulence factors; e) age determination of the virus in LTNP patients; f) studies on the prevalence and incidence of infection with hepatotropic viruses; and g) functional studies on the HIV envelope and the modulation of CD4 induced by HIV infection and their implications in viral pathogenicity.

The collection of the samples from HIV-infected patients is located at the beginning of the chain of translational research[6]. To carry out quality research that could eventually end in a personalized treatment for HIV, it is fundamental to guarantee the availability, quality and traceability under a strict system of quality management of samples obtained through standardized techniques that can be used in studies on AIDS. Another important characteristic of the BioBank is that it can generate unlimited quantities of genetic material from patients, allowing the use of these samples indefinitely[7]. Moreover, research projects can be performed readily with samples for this HIV BioBank. The provision of this material is free and only depends on the quality of the research project that must meet the ethical and legal criteria established by the BioBank.

The number of samples stored in a BioBank is another relevant aspect required to support the needs of personalized medicine. Therefore, only a large number of samples can provide a sufficient size of specific patients subgroups to be useful for evaluating individual variations of disease[6]. The excellent traceability of the samples of the distinct HIV cohorts gives investigators the possibility to study subgroups of HIV-patients which are well characterized in distinct stages of the disease and to correctly carry on the evolution of the infection in a group of individuals over a long time period. Furthermore, the large quantity of samples that are available allows researchers to conduct studies with higher scientific and statistical significance. However, it should not be forgotten that these multidisciplinary tasks can only be fully exploited thanks to the generous donors, who rightfully expect progress to be made in HIV infections using their donations. Therefore, sharing data and samples for good research need to be an obligation for those involved in decisions on access of donor samples. The manager of the sample resource as well as others involved in the decision on the access to the samples in the HIV BioBank are merely the caretakes of the samples and must act in the best interest of the donors.

Therefore, to exploit the full potential of the HIV BioBank, networking between individual biobanks is indispensable[3]. As a requirement for international cooperation, it is not only necessary to define common standards for sample quality and data formats[8], but also to consider the differences in ethical, legal and social environments in the different countries of partner biobanks[9,10]. Moreover, BioBank management and governance need to cover a variety of aspects such as compliance with biosafety and biosecurity regulations, as well as keep a balance between sample use and accrual. The HIV BioBank has been created to offer an important support to the global research in the HIV infection. Its success is measured as much in publications as in technical and scientific advances achieved via the use of these samples.

## Competing interests

The authors declare that they have no competing interests.

## Authors' contributions

IG and NdC: had primary responsibility for all protocol development and implantation of system of quality management based on the rules contained in UNE-EN-ISO 9001:2000 in the HIV BioBank.

JLJ: participated in the implantation of system of quality management based on the rules contained in UNE-EN-ISO 9001:2000 in the HIV BioBank and he had primary responsibility in the equipment management protocols.

JG, CG, CP, MJS and RL: participated in the implantation of system of quality management based on the rules contained in UNE-EN-ISO 9001:2000 in the HIV BioBank and had primary responsibility in sample management and final products management protocols.

MAMF had full access to all the protocols in the implantation of system of quality management based on the rules contained in UNE-EN-ISO 9001:2000 and supervised its design and execution, performing the final analyses, writing the manuscript. has seen and approved the final version; has final responsibility for the decision to submit for publication.
